# A High-Throughput Phenotyping System Using Machine Vision to Quantify Severity of Grapevine Powdery Mildew

**DOI:** 10.34133/2019/9209727

**Published:** 2019-08-25

**Authors:** Andrew Bierman, Tim LaPlumm, Lance Cadle-Davidson, David Gadoury, Dani Martinez, Surya Sapkota, Mark Rea

**Affiliations:** ^1^Lighting Research Center, Rensselaer Polytechnic Institute, Troy, NY 12180, USA; ^2^United States Department of Agriculture-Agricultural Research Service, Grape Genetics Research Unit, Geneva, NY 14456, USA; ^3^Plant Pathology and Plant-Microbe Biology Section, School of Integrative Plant Science, Cornell University, Geneva, NY 14456, USA

## Abstract

Powdery mildews present specific challenges to phenotyping systems that are based on imaging. Having previously developed low-throughput, quantitative microscopy approaches for phenotyping resistance to* Erysiphe necator* on thousands of grape leaf disk samples for genetic analysis, here we developed automated imaging and analysis methods for* E. necator* severity on leaf disks. By pairing a 46-megapixel CMOS sensor camera, a long-working distance lens providing 3.5× magnification, X-Y sample positioning, and Z-axis focusing movement, the system captured 78% of the area of a 1-cm diameter leaf disk in 3 to 10 focus-stacked images within 13.5 to 26 seconds. Each image pixel represented 1.44 *μ*m^2^ of the leaf disk. A convolutional neural network (CNN) based on GoogLeNet determined the presence or absence of* E. necator* hyphae in approximately 800 subimages per leaf disk as an assessment of severity, with a training validation accuracy of 94.3%. For an independent image set the CNN was in agreement with human experts for 89.3% to 91.7% of subimages. This live-imaging approach was nondestructive, and a repeated measures time course of infection showed differentiation among susceptible, moderate, and resistant samples. Processing over one thousand samples per day with good accuracy, the system can assess host resistance, chemical or biological efficacy, or other phenotypic responses of grapevine to* E. necator*. In addition, new CNNs could be readily developed for phenotyping within diverse pathosystems or for diverse traits amenable to leaf disk assays.

## 1. Introduction

Phenomics is revolutionizing plant phenotyping with high-throughput, objective disease assessment. In particular, machine vision approaches have enabled rapid progress in trait analysis under controlled conditions, including the analysis of quantitative trait loci for host resistance [1]. At its simplest, machine vision involves image capture and image analysis, both of which can be automated for higher throughput. Applied to plant disease quantification, image capture approaches have included batch imaging with a smartphone [2], flatbed scanner [3], or multispectral imager [4], among other devices. Image analysis approaches range from processes that result in pixel counting metrics, as in the above cases, to algorithms for detection of complex structures [5].

Classification algorithms are an area of machine vision that has experienced tremendous growth over the past decade with the development of convolutional neural networks (CNNs), a form of artificial intelligence that is loosely based on the neural architecture of animal visual systems [6, 7]. For a description of CNNs and recent advances in machine vision the reader is directed to review articles on this topic [8, 9]. Recent advances in deep learning CNNs have brought their performance to levels that rival human observers for correctly classifying labeled images. CNNs have been successfully applied to many biological classification problems including the classification of leaf images for species identification and the detection of different diseases and stresses [10–12].

Of particular significance to this study, Google® researchers developed a competition-winning network in 2014 called GoogLeNet [13] that successfully classifies images depicting English language nouns from the ImageNet database [14]. GoogLeNet is available as freeware for others to use and adapt to their own purposes. Through a process called transfer learning, a neural network trained to classify images according to one set of outcome categories (e.g., English language nouns) can be retrained to classify images according to a different set of outcome categories (e.g., disease symptoms). Because training a network from scratch is a computationally intensive process that requires a large set of labeled inputs, transfer learning can improve the performance of large CNNs where there are limited training data and computational resources. Such is the benefit of starting with GoogLeNet, a CNN trained using over one million images where the weights and offsets describing the filters and neural interconnects of the network start at values that extract features that work well for classifying a diverse set of different shapes, textures, colors and patterns. Retraining GoogLeNet can be relatively quick compared to training from scratch (hours instead of days or weeks), using a relatively small training set (thousands as compared to millions of images) specific to the task at hand.

Powdery mildews present specific challenges for imaging and machine vision, especially in the earliest stages of development. In live specimens viewed at relatively low magnifications (i.e., 5–30×), hyphae appear transparent and are closely appressed to a leaf surface [15] overlain by a topographically complex and highly reflective wax cuticle prone to emit glare when live specimens are illuminated for microscopy and photomicrography. With appropriate lighting or staining, nascent colonies originating from conidia or ascospores of grapevine powdery mildew (*Erysiphe necator*) can be resolved using 3–10× magnification within 48 hours after inoculation. The fungal hyphae are approximately 4–5 *μ*m in diameter, hyaline, tubular and superficial on the leaf surface [15]. They produce lobate organs of attachment and penetration (appressoria) at regular intervals. Except for the absorptive haustoria within the living host epidermal cells subtending the appressoria,* E. necator* is wholly external to the host. Once sufficient host tissue is colonized (generally within 5 to 7 days after inoculation), the colony becomes sporulation-competent and synchronously produces upright conidiophores over much of the colony surface. These upright conidiophores and the chains of conidia that they bear lend the macroscopically powdery appearance to the colony for which the pathogen group is named.

Grapevine powdery mildew caused by* E. necator* presents a significant management challenge everywhere grapes are grown. For example, powdery mildew management in California accounts for 9% to 20% of cultural costs for grape production, primarily from fungicide applications [16], as nearly all cultivated* Vitis vinifera* grapevines are highly susceptible. As a result, a major effort is underway to genetically map host resistance loci for introgression from wild* Vitis* into domesticated* V. vinifera* [17–20]. In previous studies of host resistance to* E. necator* and pathogen resistance to fungicides, controlled phenotyping of* E. necator* on grape leaf tissue used 1-cm diameter circular leaf disks cut from living grape leaves, arrayed on agar within Petri dishes or glass trays [21–23]. For host resistance assessment at 2 to 10 days after inoculation, the disks were destructively sampled by bleaching the leaf samples and then staining with a dye to make the hyphae more visible for phenotypic analysis under brightfield microscopy at 100× to 400× [21]. Severity of infection was estimated by hyphal transects, a point-intercept method adapted from vegetation analysis, where the number of hyphal interceptions of axial transects in the field of view was recorded as the response variable. These hyphal transect interceptions have proven to be an effective means of quantifying disease severity in large experiments to detect quantitative trait loci (QTL) in segregating populations [21]. The high magnification (400×) required by human observers to accurately assess and quantify hyphal growth, and the resultant shallow depth of focus (2 *μ*m) and small field of view (0.045 cm) makes the foregoing a relatively slow process. For example, obtaining accurate assessments of hyphal growth in experiments involving 1600 leaf disks required approximately 20 to 60 person-days of microscopic observation.

Parallel to advances in CNNs, the pixel density now available in highly sensitive, high-resolution CMOS sensors used in full-frame (24×36 mm) Digital Single Lens Reflex (DSLR) cameras now approaches 50 megapixels. Paired with long-working distance high-resolution optics, this advancement now allows the synoptic capture of nearly the total area of a powdery mildew colony borne on a 1-cm leaf disk in a single high-resolution image. Focus-stacking algorithms can now rapidly assemble a fully focused image from stacks of partially focused images representing optical sections of a specimen to accommodate the complex topography of a leaf surface. The capacity to rapidly collect high-resolution and fully focused images of a 1-cm diameter area (compared to 0.045 cm under 400× microscopy) strengthens the case for machine vision, which could then process the images far more rapidly than a human observer. The goals of our study were to develop an Automated Phenotyping System (APS) that could

- image at a rate of 1600 leaf disk samples per 8-hour day to provide the throughput required for QTL analysis;
- nondestructively analyze and track progression of pathogen growth over several days;
- quantify severity with a level of accuracy similar to that of trained human observers, with a metric that correlates well with counts from the standard hyphal transect technique.

## 2. Materials and Methods

### 2.1. Experimental Design

Three unreplicated experiments were undertaken to evaluate the performance of the APS and demonstrate its capabilities.

  Experiment 1: Expert comparisons.
  Experiment 2: Time-series mapping of growth.
  Experiment 3: Comparison to hyphal transect technique.

#### 2.1.1. Plant and Pathogen Material

Isolate NY90 of* E. necator* was used in all experiments except full-sibling progeny 452033, 452036, and 452051 described below. For these three samples, Musc4 was used in the time-series experiment (experiment 2, described below) and NY1-137 in the hyphal transect comparison experiment (experiment 3) because these isolates were being used by the VitisGen project [24] to map resistance in that family. All isolates were previously described, and their phenotypes can be summarized by their differential virulence on RUN1 vines: avirulent NY90, fully virulent Musc4, and moderately virulent NY1-137 [18, 25]. Several grape varieties were used in the experiments described here to challenge the system with different amounts of leaf hairs and levels of susceptibility to* E. necator*, including 10 different resistance loci (Table 1). Leaf sampling and processing for phenotyping resistance to* E. necator* was done as described by Cadle-Davidson et al. [21]. Briefly, leaves were sampled from the third node of a grape shoot (these leaves are typically translucent and about half the width of a fully expanded leaf), then surface sterilized, subsampled using a 1-cm cork borer, and arrayed on 1% agar media in a Petri dish or 32 × 26 × 2 cm Pyrex® tray (adaxial surface up). Inoculum consisted of* E. necator* conidiospores (5 × 10^4^ per mL) suspended in distilled water containing 0.001% Tween-20. The leaf disks were inoculated by spraying them with an aerosol of the above suspension until the leaf surface bore visible droplets approximately 5- to 10-*μ*l in volume. The droplets were allowed to dry, then the trays were immediately covered to maintain high humidity and were incubated at 23°C for a 12-hour photoperiod with 45 *μ*mol*∗*m^−2^*∗*s^−1^ of photosynthetically active radiation (PAR) irradiance until and between imaging. Covers used to maintain humidity were removed for imaging and replaced immediately afterward.

#### 2.1.2. APS Description

*(0) Overview*. To progress from the aforementioned single-point microscopy and human observer-based methodology toward a high-throughput, repeated measures phenotyping system, an APS was developed, detailed in subsequent sections. The system paired a high-resolution DSLR camera and a long-working distance macrofocusing lens. Relatively low magnification (3.5×) and long-working distance (5 cm) of the optical system resulted in a depth of focus of 200 *μ*m compared to the 2 *μ*m depth of focus obtained at 400× in the human-based system. This allowed the system to image the entire disk synoptically in 3 to 10 focus-stacked images. The stacked images were assembled into a single fully focused image through a focus-stacking algorithm [26].

**Figure 1:**
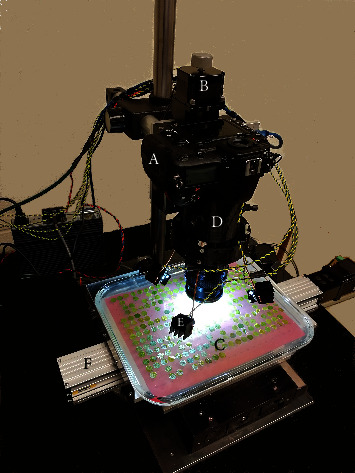
*Assembled system for image capture*. A Nikon model D850 46 MP digital SLR camera and 60mm F/2.8 D Micro Autofocus lens (A) were suspended from an automated robotic Z-axis positioner (B) above a sample tray (C) carrying 1-cm leaf disk samples arrayed in a 22 by 15 matrix. The lens was stabilized by an accessory collar (D) that also bore the four white LEDs (E) that illuminated the samples. The tray was supported on an automated robotic stage (F) to provide movement in the X and Y planes.

To move from one sample to the next, the APS used an X-Y motorized stage (Figure 1(F)) to move a tray (Figure 1(C)) holding up to 330 1-cm grape leaf disks beneath the camera (Figure 1(A)), and a computerized and integrated control and image analysis system to capture high-resolution images of nascent* E. necator* colonies at high speed. The grape powdery mildew pathosystem was used as a model to assess changes in disease severity in the context of a grape breeding project screening diverse* Vitis* germplasm across North America [24]. The APS enabled live imaging and processing an entire tray without operator intervention. With the tray resting on a two-axis translation stage, samples were automatically moved into position for imaging. Important characteristics of the positioning and camera mounting system include agile movement across different focusing planes for dynamic depth-of-field enhancement, stability, minimal vibrations that are quickly damped after movement and quick sample-to-sample movements to help meet our throughput goal. The images were analyzed for infection after being saved.

*(1) Positioning and Imaging Hardware*. Three linear actuator stages were orthogonally arranged to provide the camera with three axes of positioning movement. The range of motion of the X and Y axes provided a working sample area measuring approximately 20 × 30 cm. The Z-axis had 5 cm of travel for finding focus and generating a stack of images for the enhanced depth-of-field image processing that was employed [27, 28]. All stages were controlled by a program written in MATLAB® 2017B [29]. Stepper motors were driven using trapezoidal velocity profiles, accelerations of 1250 and 10 mm·s^−2^ for the X and Y axes, respectively, and maximum velocities of 50 and 8.75 mm·s^−1^. The Z-axis had an asymmetrical acceleration/deceleration of 55 mm·s^−2^ and -20 mm·s^−2^ to decrease settling time when stopping. The maximum velocity of the Z-axis was 55 mm·s^−1^.

**Figure 2:**
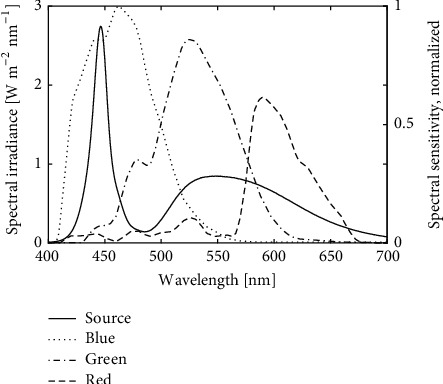
*Sample illumination and camera sensitivity*. Measured spectral irradiance of grape leaf disks (solid black line) as samples were illuminated by four phosphor-converted Indium Gallium Nitride (InGaN) “white” light emitting diodes. The spectral matching between the illuminant and the camera is compared to the reported spectral sensitivity of the red, green, and blue channels (dashed, dot-dashed, and dotted curves, respectively) for an RGB CMOS sensor [30] similar to that of the Nikon model D850 camera used in the present study.

The system paired a DSLR camera with a 46 MP 24 × 36 mm CMOS sensor (Nikon D850, Figure 1(A)) and a long-working distance macrofocusing lens (Nikon Nikkor 60mm F/2.8 D Micro autofocus with four PK-12 extension tubes) with a RGB color registration filter (Figure 2, [30]). This configuration obtained 3.5× magnification and a 1.0 × 0.67 cm field of view. At this magnification each square image pixel represents 1.44 *μ*m^2^ ((1.0 cm per image length/8256 pixels per image length)^2^). A custom-designed 3-D printed support for the camera lens tube was used to stabilize the assembled lens and extension tubes (Figure 1(D)). The lens support also contained provisions for mounting the four LEDs (Figure 1(E)) which were supported on 75-mm lengths of 2-mm diameter copper wire. The illumination angle was approximately 80 degrees with respect to the sample surface normal. The light sources were phosphor-converted cool white LEDs (CREE XML2-W318) with direct emission peaking at 446 nm (Figure 2) coupled to narrow-spot collimating lenses. These LEDs provided an irradiance of 170 W·m^−2^ (50,000 lux) on the leaf sample measured by a spectroradiometer (Photoresearch model PR740) viewing a white reflectance standard (Labsphere, model SRT-99-050). The shutter speed was 1/500 seconds with an ISO setting of 1000.

*(2) Image Capturing*. The sample tray was positioned against corner guide rails on the stage platform for accurate and repeatable placement. Even though the samples were placed on a grid, they might not be fully centered in the image, and the placement of the grid might differ from tray to tray. Therefore, we developed a procedure implemented in software to find the approximate center of a sample and move it to the center of the image. This process was repeated until the change in position became sufficiently small, or the program had iterated 10 times.

Due to the irregular surface of a leaf sample (±500 *μ*m or more), and the magnification needed to resolve the hyphae, the limited depth of focus of the lens system (approximately ±100 *μ*m) would not be able to bring the whole sample into focus. Instead, multiple images at varying focus heights around the center image focus were taken so that when combined using an image stacking software program, most if not all of the processed image was well focused. We devised an automated procedure to determine appropriate focus heights. Depending on the variations in sample height, three to ten images were then taken at different focus heights using the maximum camera resolution (8256 × 5504 pixels). Helicon Focus 6 [26] software was used to stack the images using the “Method C” setting which Helicon specifies as being the most useful for images with multiple crossing lines and complex shapes, but with the potential for increased glare in an image [31]. The processed images were saved for offline analysis using computer vision to detect and quantify hyphae.

*(3) Image Analysis*. The approach taken to determine the amount of infection in a leaf sample was to divide the image into an array of smaller subimages and then classify each subimage as either containing hyphae or not. Each subimage measured 224 x 224 pixels yielding 864 nonoverlapping, adjacent subimages per leaf disk image. The amount of hyphae present in the whole image was then estimated by the percentage of subimages containing hyphae. This formulation of the problem yielded a quantitative measure of infection from binary image classifications.

We modified GoogLeNet from the MATLAB® Deep Learning Toolbox, version 18.1.0, to be a two-output classifier (subimage infected or not infected). Each subimage was 224 × 224 pixels to match the input layer dimensions of GoogLeNet without resizing. The last three network layers of GoogLeNet were removed and replaced by three new layers: (1) a 2-neuron fully connected layer, (2) a softmax layer, and (3) a classification layer. Other than the three modified network layers the network weights and offsets were initialized to the pretrained values in the distributed ImageNet version. Initialization values for the three new layers were randomly chosen from a Gaussian distribution with zero mean and standard deviation 0.01 for the weights and zero for the offsets. We named this new network “GPMNet” for grapevine powdery mildew modification of GoogLeNet.

The training dataset consisted of 14,180 subimages from 19 whole leaf disk images. Only subimages that contained at least 90% leaf surface by area were used for training. The training subimages were generated from four categories of leaf disk images, each representing one of three varieties: Chardonnay (young and old leaves), Bloodworth 81-107-11 and* V. cinerea* B9. These samples exhibited a range of different characteristics including different amounts of leaf hairs, color differences, and texture differences (e.g., glossy/dull, smooth/rough). Two authors, AB and TL, independently labeled the training set subimages; AB provided roughly 75% of the labels. A separate independent dataset was collected for validating the CNN as described in the Performance Evaluations section. Training was done using MATLAB® Neural Network Toolbox™ [29] with GoogLeNet add-on package. The following hyperparameters were used for training:

- Solver type: Stochastic Gradient Descent
- Initial learning rate: 2×10^−4^
- Learning Rate Schedule: piecewise (decreases by a factor of 0.63 every epoch), learning rate multiplier of 3 for the added fully connected layer
- Momentum: 0.9
- L2 Regularization factor (weight decay): 0.0001
- Batch size: 32
- 70/30 split of the 14180 subimages randomly assigned into groups of training/validation datasets
- Training set augmentation: 3× by including copies of the subimages that were flipped horizontally and vertically about the image centerlines

Training stopped when the cross entropy of the outcome and known responses of the validation set stopped decreasing by meeting the criterion of 20 direction reversals when computed once every 1600 image iterations. The image analysis software is available at https://github.com/LightingResearchCenter/GPMNet.

#### 2.1.3. Performance Evaluations

*(1) Experiment 1: Expert Comparisons*. New samples from four varieties of grape were selected based on resistance to* E. necator*: susceptible Chardonnay, moderately resistant DVIT2732-6, and highly resistant DVIT2732-9 and DVIT2732-81. Images taken 3 days and 9 days after inoculation (dpi) were included for the low and moderately resistant varieties, while only 9 dpi images were included for the highly resistant varieties because there was no change over time in the infection state for these highly resistant varieties. The six images were distributed to members of the research team (AB, TL, and SS) experienced in identifying hyphae. A custom application was programmed in MATLAB® to display 224 × 224 pixel subimages and record the experts' responses of whether the subimages contained hyphae or not. In addition to showing a subimage and response buttons, the program displayed a second window showing the whole leaf disk with the subimage demarcated with a red outline. This second image could be panned and zoomed to allow the person classifying the subimage to see the image in the context of the whole leaf disk. The same leaf subimages, approximately 800 per leaf disk, were classified by both humans and the CNN.

Statistical Analyses: Percent agreement, calculated as (true positives + true negatives)/(number of images), was calculated for all pairs of experts and the APS. The correlation (Pearson's r) was calculated among the different experts and the APS.

*(2) Experiment 2: Time-Series Mapping of Growth*. Three sets of three grape varieties were selected: highly susceptible Chardonnay with three replicate leaf disks (here named as 165-Chardonnay-t1, 165-Chardonnay-t3 and 330-Chardonnay); unreplicated moderately resistant full-sibling progenies from the biparental cross “Horizon” ×* V. rupestris* (here named as 24-452033, 27-452036 and 38-452051); and unreplicated highly resistant Ren-stack progeny containing RUN1, REN1, REN6, and REN7 genes (here named as 157-Ren-stack and 316-Ren-stack). The leaf disks were imaged and analyzed by the automated system once per day on days 2, 4, 6, and 9 after inoculation.

Statistical Analyses: Area under the disease progress curve (AUDPC) was calculated by the simple midpoint (trapezoidal) rule [32].

*(3) Experiment 3: Comparison to Hyphal Transect Technique*. After imaging the leaf disks of experiment 2 at 9 days after inoculation, the leaf disks were bleached, stained and the state of infection was quantified by the hyphal transect method, which quantifies the number of times an imaginary vertical and horizontal transect is crossed by hyphae [21].

Statistical Analyses: Comparisons between the hyphal transect technique and the APS results were evaluated by R^2^ values modeling the APS percent infected subimages as a linear function of the hyphal transect count. The hyphal transect count was also compared to the AUDPC from 2 to 9 dpi (Exp. 2), and to the growth rate coefficient for a simple logistic population growth curve, originally proposed by Pierre-François Verhulst in 1838, using R^2^ for a linear model.

## 3. Results

### 3.1. Image Capture and Throughput

**Figure 3:**
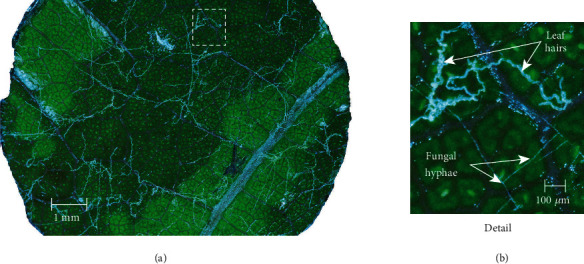
*Full resolution (46 megapixel) image produced through focus-stack processing of images captured in the Z-plane*. (a) Leaf disk sample imaged 3 days after inoculation with* Erysiphe necator* conidiospores. Illumination of the live sample at near-grazing angles revealed detail of the hyaline hyphae without excessive glare from the highly reflective leaf cuticle. (b) Detailed area of image illustrating morphology of fungal hyphae and nearby leaf trichomes (hairs).

Based on informal evaluation, an illumination angle of roughly 80° measured from the surface normal was practically achievable to provide high contrast of hyphae with low background illumination of the leaf surface while minimizing shadows and not interfering with adjacent samples. The chosen magnification allowed for approximately 78% of leaf disk area to be captured in a single focus-stacked processed image while still resolving hyphae with high contrast (Figure 3). The time needed to image each leaf disk varied depending on the flatness of the leaf disk which in turn affected the focusing and number of focus-stack images needed. Times typically ranged from 13.5 to 26 seconds (Table 2). Thus, between 1100 and 2100 images could be collected in 8 hours depending on the flatness of the samples, resulting in a single focus-stack-processed image in 24-bit tiff format, 8256 × 5504 pixels, for each leaf disk.

### 3.2. Neural Network Training Results

**Figure 4:**
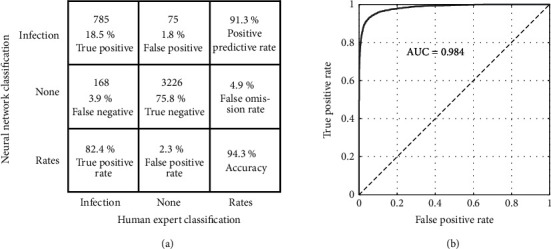
*Confusion Matrix and Receiver Operating Curve*. Confusion Matrix (a) and Receiver Operating Curve (b) for GoogLeNet retraining outcome assessment for the training validation data set

**Figure 5:**
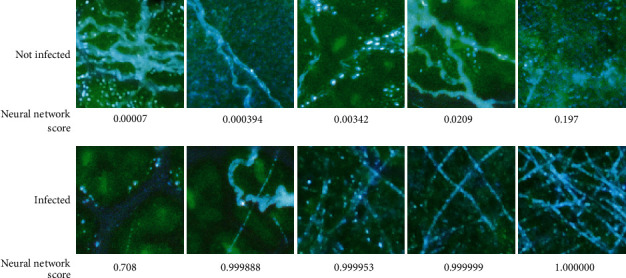
*Leaf disk subimages classified by a human expert along with corresponding neural network scores*. Subimages of leaf disks classified by a human expert as either free of visible signs of infection (Not infected) or containing hyphae of* Erysiphe necator* (Infected), along with corresponding neural network scores.

Retraining of GoogLeNet required 3.4 hours of computation time using an Intel Xeon CPU E31225 at 3.1 GHz with an Nvidia GeForce GTX 1050 Ti GPU and iterated through the set of 9920 training images (70% of labeled dataset) 32 times. The resulting CNN had a classification accuracy of 94.3% and ROC area under curve of 0.984 (Figure 4) for the validation subset of the training images for correctly classifying the subimages as infected or not (Figure 5). This accuracy is based on a criterion set at 0.5 on a scale from zero to one, but the criterion could be set to other levels depending on the desire to either reduce false-positive responses (higher criterion) or increase sensitivity by reducing false negatives (lower criterion).

**Figure 6:**
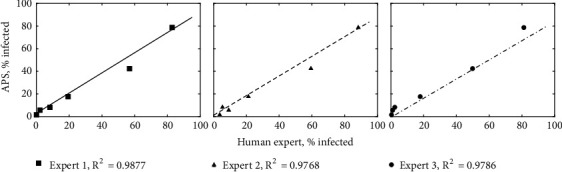
*Relationship between severity of infection as determined by the Automated Phenotyping System (APS) neural network versus three independent human experts*. Human experts rated the same subimages analyzed by the neural network, and results were analyzed by linear regression. The coefficients of correlation of neural network scores and human observer scores were 0.9877, 0.9768, and 0.9786 respectively for Experts 1, 2, and 3.

*Experiment 1: Expert Comparisons*. The three experts and the neural network were in agreement for 89.3% to 94.8% of subimages (Table 3). As expected,* E. necator* hyphae were rarely detected in resistant leaf disks at 9 dpi (no more than 3.7% of subimages), and moderately resistant DVIT2732-6 was intermediate between susceptible Chardonnay and the two resistant samples (Table 4). As operated with a false-positive rate of 2.3%, the neural network was slightly less sensitive than human observers in detecting hyphae as given by the slope of the linear trend line being 0.87 (Figure 6). The correlation between experts and the APS was 0.977 or greater for all pairings (Table 5). The highest correlations between experts and the APS were for Expert 1 (AB), followed by Expert 3 (SS), while the highest agreement with the APS was Expert 1 followed by Expert 2 (TL).

**Figure 7:**
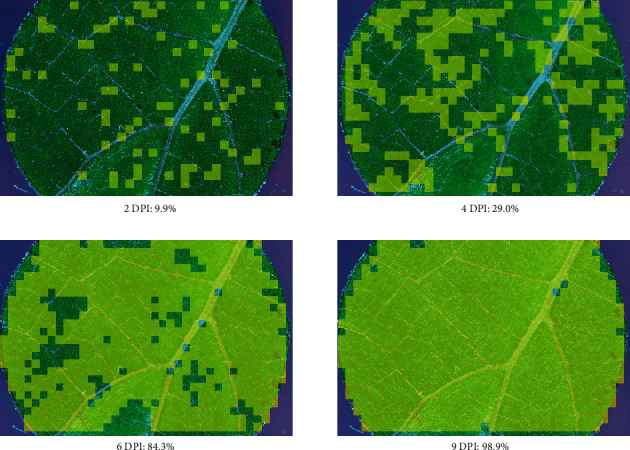
*Leaf disks imaged and scored for disease severity*. A leaf disk of 452036 (“Horizon” ×* V. rupestris* B38) inoculated with* Erysiphe necator* isolate Musc4 was imaged and scored for disease severity successively at 2, 4, 6, and 9 days after inoculation (DPI). The images are overlaid with lighter shade to show subimages that were classified as infected (score > 0.50). The total percentage of infected leaf disk area, calculated as the percentage of subimages classified as infected, is given below each image.

**Figure 8:**
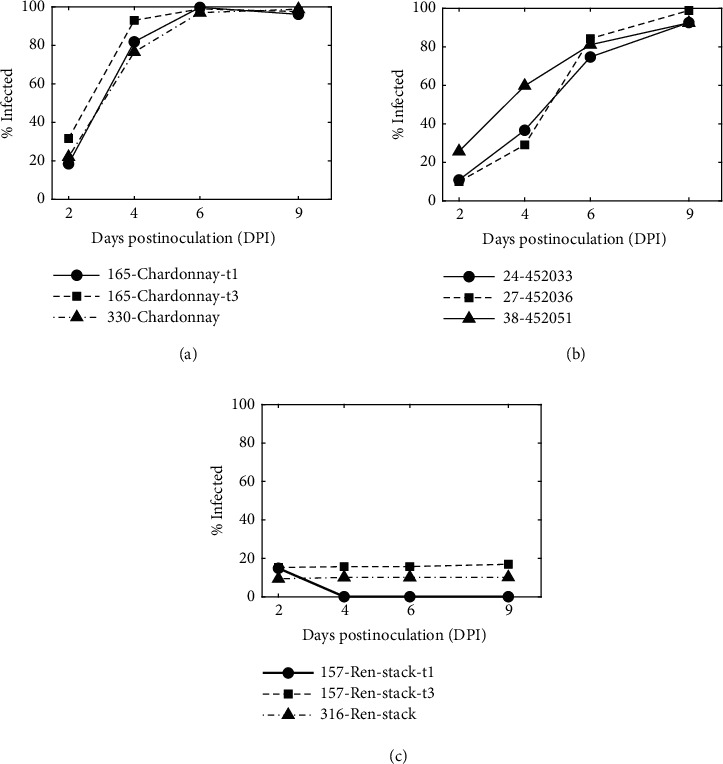
*Neural network determination of leaf disk infection as a function of time after inoculation*. (a) Susceptible varieties, (b) moderate varieties, and (c) resistant varieties are shown here. In the figure legend, disk number relates each sample to the sample names shown in Table 5. Replicate disks t1 and t3 were from the same vine and were incubated and imaged on two different trays.

*Experiment 2: Time-Series Mapping of Growth*. From 2 to 9 dpi, the percentage of subimages with* E. necator* on susceptible or moderate samples increased along a logarithmic or sigmoidal curve, saturating near 100% at 6 or 9 dpi, respectively (e.g., Figure 7), while detection on resistant samples did not increase (Figure 8).

*Experiment 3: Comparison to Hyphal Transect Technique*. R^2^ values modeling time-series outcomes as a linear function of hyphal transect counts were stronger for growth rate (R^2^ = 0.933, p < 0.001) and AUDPC (R^2^ = 0.951, p < 0.001) than for percent of infected subimages at 9 dpi (R^2^ = 0.867, p < 0.001; Table 6).

## 4. Discussion

In this study, we developed an APS system capable of imaging 1100 to 2100 leaf disks in an 8-hour workday, passing those images to a CNN capable of accurately detecting the presence or absence of* E. necator* hyphae in each of 800 subimages per disk, and capable of capturing time-course data. With this throughput and accuracy, which represents a 20- to 60-fold increase in throughput over manual assessment, our APS can now be implemented for phenotyping* E. necator* growth for various research applications, including host resistance, fungicide resistance, and other treatment effects. While CNNs have been previously applied to macroscopic images of plant leaves for disease assessment [e.g., [33]], and even for quantitative assessment of the severity of powdery mildew infection [34], to our knowledge our system is the first to apply CNN techniques to the microscopic detection of fungal hyphae before sporulation occurs. Microscopic detection of hyphae enables early detection of infection and growth rates, which increases capabilities in testing for treatment effects, such as host resistance or other disease management strategies.

The goal of the system is to have automated scoring that is highly correlated with human observers assessing the severity of infection. The outcome was much better than correlations previously obtained (r = 0.43 and 0.80) in a leaf disk-based computer vision system using a smartphone and pixel counting to quantify downy mildew caused by* Plasmopara viticola* [2] and similar (r = 0.94) to a flatbed scanner and pixel counting used to quantify* Septoria tritici* blotch caused by* Zymoseptoria tritici* [3]. The agreement between experts and the APS reflects the amount of training set classifications provided by each; experts supplying more training data had higher agreement with the APS. However, correlations between experts and the APS did not strictly follow this ordering. Agreement among the experts was higher than agreement between any of the experts and the APS, suggesting that any bias introduced by which expert to use for CNN training was small. However, the accuracy among experts was only slightly higher than that between experts and the APS suggesting that new methodologies would need to be developed replacing human experts to assess further improvements in AI performance without observer biases.

The datasets used for evaluating GPMNet were acquired after the training dataset and included different grape germplasm, although Chardonnay was included in both. This approach ensured that the testing dataset was independent of the training dataset and perhaps provided a more rigorous test than randomly dividing a single image database into training, validation and test images as is commonly done [10–12]. Having a CNN that generalizes well to new accessions reduces the need to continually retrain the CNN, and is an important consideration when working with diverse breeding germplasm with a broad set of grape leaf characteristics.

An instance of different germplasm challenging the generality of the CNN is the higher than expected false-positive rate (10–16%) for the resistant samples 157-Ren-stack and 316-Ren-stack (Table 6) compared to the false-positive rate for the training data (2.3%). While this is likely a CNN generalization issue, other differences in the experiment execution (such as quantity of viable inoculum applied) or sample images (such as the lighting or image focus), can also negatively affect the results. As a case in point, sample 157-Ren-stack-t1 exhibited a decrease in infection on day 4 and thereafter. Inspection of the images revealed that starting on day 4 roughly half the image was not in sharp focus, probably due to the leaf bending up off the agar along a major vein by a distance more than could be accounted for by the focus-stacking process. Whatever caused the false-positives in the sharply focused images was no longer present in the blurred images. A way to mitigate the training generalization problem is to make the training image set as inclusive as possible [35], which in this case means representative of the different grape samples that will be later analyzed.

Optical techniques for making hyphae more visually prominent are limited, which likely explains why previous image-based phenotyping usually required destructive sampling and staining [5]. Despite these imaging limitations, CNN-based machine vision systems can produce results similar to destructive sampling techniques without the need for staining and with much greater throughput. However, a large part of the success of the APS is in achieving high-resolution, high contrast images. Highest contrast of hyphae against the leaf background is attained by illumination at a high angle of incidence on the sample. Presumably, this is due to the 3-D structure of the hyphae intercepting the light and redirecting it to the imaging lens, while providing relatively ineffective illumination of the leaf surface itself.

Choosing an illuminating spectrum that minimizes reflection from the leaf surface also increases the contrast of the hyphae against the leaf background. Leaf reflectance is lowest for wavelengths less than 460 nm [36]. As with most biological tissues, the scattering coefficient of the hyaline hyphae can be expected to increase with shorter wavelengths [37], thereby increasing their visibility as the illuminating wavelength is decreased. Considering both effects, a light source having significant spectral output circa 450 nm can increase the brightness of the hyphae in the image while keeping the surrounding leaf surface dim. While even shorter wavelength illumination could further enhance this effect, the silicon-based image sensors in commercial cameras, which employ red, green, and blue sensor channels (RGB), rapidly lose sensitivity for still shorter wavelengths and image quality degrades as commercial optics are not optimized for wavelengths shorter than approximately 430 nm. Thus, with appropriate lighting, as employed by the APS system, the 4–5 *μ*m in diameter fungal hyphae in nascent colonies of* E. necator* can be resolved on live samples, using 3× magnification within 48 hours after inoculation.

The hyphal transect method represents the previous gold standard for manual quantification of grapevine powdery mildew disease severity [21] and aside from throughput and repeated measures, there are strengths and weaknesses in data quality compared to the APS developed here. The primary weakness of hyphal transects comes from subsampling only along the vertical and horizontal transects, thus missing any fungal growth that occurs away from these lines. In the APS, once hyphae are present in nearly every subimage the neural network metric saturates at 100%; however, hyphal transect counts can continue to increase as the density of hyphae increases. Thus, if a graded response among susceptible individuals is important, APS data need to be analyzed sooner after inoculation. The CNN could be modified from detecting presence of hyphae in subimages to also estimating the number of hyphae in subimages. A smaller subimage size could potentially better reveal hyphae density differences, but smaller subimages provide less information for determining an accurate classification, so this approach would have limited applicability.

Another approach to improving the correlation between APS results and the hyphal transect method is to use the time-series data to mathematically model fungal growth and predict hyphal transect counts at time points after inoculation. Susceptible samples showed rapid growth saturating near 100% infected area by day 6, while moderate samples showed delayed exponential growth saturating near day 9. These examples demonstrate the utility of the time-series data for providing growth information, even for the small sample size presented here. These or other time-series analyses, such as area under the disease progress stairs [32], may more accurately describe disease progress in other datasets.

While we chose GoogLeNet for the current study, more recent image classification networks are available (e.g., Inception-V3 [38] or ResNet [39]) that have surpassed GoogLeNet for accuracy in classifying labeled images, but versions of these networks are often much larger than GoogLeNet, thus requiring more time and computer resources to train and utilize. Meanwhile, their efficiency in terms of accuracy per computational unit can be significantly lower; to the point where computation time is so large that it limits sample throughput [40]. To verify the lower efficiency of larger networks on our classification problem we tried several training runs using an Inception-V3 network, modified similarly to how GoogLeNet was modified to fit our needs and using the same training dataset. Inception-V3 had at most a 1% increase in training set accuracy but required twice the computation time.

## Figures and Tables

**Table 1:** *Plant Material*. The resistance locus status of grapevine germplasm samples used for neural network training and the three performance experiments: (1) expert comparison, (2) time series, and (3) hyphal transect comparison.

Expected response	Sample ID*∗*	Expected resistance loci †	Task
Resistant	**Ren-stack**	*RUN1*, *REN1, REN6*, and *REN7*	Experiments 2 and 3
Resistant	DVIT**2732-9** and DVIT**2732-81**	*REN4*	Experiment 1
Moderate	DVIT**2732-6**	unknown	Experiment 1
Moderate	*Vitis cinerea *B9	*REN2*	Training
Moderate	**452033**, **452036**, and **452051**	*REN3/REN9* or similar	Experiments 2 and 3
Moderate	Bloodworth 81-107-11	*RUN2.1*	Training
Moderate	*V. vinifera* “Chardonnay” old	Ontogenically resistant leaves	Training
Susceptible	*V. vinifera* “**Chardonnay**”	*SEN1* susceptibility	Training, Experiments 1, 2 and 3

*∗* The bold terms are used in the remainder of the text, tables, and figures for simplicity.

† The resistance loci (alleles) present in each vine are listed here, based on AmpSeq analysis of previously published markers [17–20]. DVIT2732-6 lacks REN4 but has moderate resistance from an unknown pollen donor. The full-sibling progeny (452033, 452036, and 452051) of the biparental cross “Horizon” × *V. rupestris* B38 likely carries the *REN3/REN9 *locus conferring moderate resistance.

**Table 2:** Image capture throughput for obtaining a composite, Z-stacked leaf disk image (total), and a breakdown of the steps involved.

Task	Time required per leaf disk sample
Move to center of sample	0.5 seconds
Focus on the center of the image	3 to 4 seconds
Determine Z-stack focusing range	2.5 to 4 seconds
Move Z-axis and capture images	2.5 seconds per image, typically 3–7 images
*Total*	*13.5 to 26 seconds*

**Table 3:** Agreement, calculated as (true positives + true negatives)/(number of images), between different experts and between experts and the neural network for classifying as infected or not infected for 4000 subimages from 6 different *Erysiphe necator* inoculated leaf disk samples (given in Table 4).

% Agreement	Expert 1	Expert 2	Expert 3	GPMNet
Expert 1	100	94.8	92.9	91.7
Expert 2	94.8	100	92.3	91.0
Expert 3	92.9	92.3	100	89.3
GPMNet	91.7	91.0	89.3	100

**Table 4:** Image assessments of *Erysiphe necator* infection by different human experts and the APS (GPMNet) in terms of the percent of subimages containing hyphae at 3 or 9 days postinoculation (dpi).

Grape variety	3 dpi		9 dpi	
DVIT2732-6	Expert 1	2.4%	Expert 1	49.9%
	Expert 2	8.2%	Expert 2	56.8%
	Expert 3	5.4%	Expert 3	59.4%
	GPMNet	8.2%	GPMNet	55.4%

Chardonnay	Expert 1	17.9%	Expert 1	81.2%
	Expert 2	19.3%	Expert 2	82.9%
	Expert 3	21.2%	Expert 3	88.3%
	GPMNet	17.6%	GPMNet	84.1%

DVIT2732-81			Expert 1	0.4%
	Expert 2	0.0%
	Expert 3	3.7%
	GPMNet	1.5%

DVIT2732-9			Expert 1	1.1%
	Expert 2	2.2%
	Expert 3	3.7%
	GPMNet	1.5%

**Table 5:** Symmetrical correlation matrix (Pearson's r) of different assessors (human experts and the APS (GPMNet)) for determining percent infection with *E. necator* for 6 leaf-disk samples as described in Table 4.

	Expert 1	Expert 2	Expert 3	Human average	GPMNet
Expert 1	1.000	0.993	0.995	0.998	0.988
Expert 2	0.993	1.000	0.991	0.997	0.977
Expert 3	0.995	0.991	1.000	0.998	0.979
Human average	0.998	0.997	0.998	1.000	0.983
GPMNet	0.988	0.977	0.979	0.983	1.000

**Table 6:** Hyphal transect counts and neural network results (% infected subimages) for 6 leaf disks measured 9 days after inoculation. Growth rate coefficient and AUDPC were calculated from data shown in Figure 8.

Sample Name	Category	Transect Count (manual)			% Infected (APS)	Growth rate coefficient	AUDPC
		H	V	H+V	%		Normalized (max = 100)
157-Ren-stack-t1 (R)	No infection	0	0	0	0.1	0.041	2.6
316-Ren-stack (R)	No infection	0	0	0	10.2	0.30	11.5
157-Ren-stack-t3 (R)	No infection	0	0	0	15.8	0.36	18.3
24-452033 (M)	Moderate	83	197	280	92.6	0.97	67.0
27-452036 (M)	Moderate	146	147	293	98.9	0.99	69.8
38-452051 (M)	Moderate	130	169	299	92.4	1.23	79.6
165-Chardonnay-t1 (S)	Severe	222	139	361	96.1	1.53	94.2
330-Chardonnay (S)	Severe	237	246	483	98.8	1.48	92.6
165-Chardonnay-t3 (S)	Severe	237	253	490	97.6	1.89	100.0

## Data Availability

All data are available upon request. Please contact the corresponding author. The imaging control software is available at https://github.com/LightingResearchCenter/Plant-Imaging-Platform. The image analysis software (i.e., the neural network) is available at https://github.com/LightingResearchCenter/GPMNet.
